# Anaplastic thyroid cancer spheroids as preclinical models to test therapeutics

**DOI:** 10.1186/s13046-024-03009-8

**Published:** 2024-03-19

**Authors:** Jiangnan Hu, Kaili Liu, Chandrayee Ghosh, Tejinder Pal Khaket, Helen Shih, Electron Kebebew

**Affiliations:** 1 Department of Surgery and Stanford Cancer Institute, Stanford University, Stanford, CA USA; 2 Stephenson School of Biomedical Engineering, University of Oklahoma, Norman, OK USA

**Keywords:** Anaplastic thyroid cancer, Tumor spheroid, Gene expression, Epithelial–mesenchymal transition, Treatment response

## Abstract

Anaplastic thyroid cancer (ATC) is the most aggressive thyroid cancer. Despite advances in tissue culture techniques, a robust model for ATC spheroid culture is yet to be developed. In this study, we created an efficient and cost-effective 3D tumor spheroids culture system from human ATC cells and existing cell lines that better mimic patient tumors and that can enhance our understanding of in vivo treatment response. We found that patient-derived ATC cells and cell lines can readily form spheroids in culture with a unique morphology, size, and cytoskeletal organization. We observed both cohesive (dense and solid structures) and discohesive (irregularly shaped structures) spheroids within the same culture condition across different cell lines. *BRAF*^*WT*^ ATC spheroids grew in a cohesive pattern, while *BRAF*^*V600E*^-mutant ATC spheroids had a discohesive organization. In the patient-derived *BRAF*^*V600E*^-mutant ATC spheroids, we observed both growth patterns, but mostly the discohesive type. Histologically, ATC spheroids had a similar morphology to the patient’s tumor through H&E staining and proliferation marker staining. Moreover, RNA sequencing analysis revealed that the gene expression profile of tumor cells derived from the spheroids closely matched parental patient tumor-derived cells in comparison to monolayer cultures. In addition, treatment response to combined BRAF and MEK inhibition in *BRAF*^*V600E*^-mutant ATC spheroids exhibited a similar sensitivity to the patient clinical response. Our study provides a robust and novel ex vivo spheroid model system that can be used in both established ATC cell lines and patient-derived tumor samples to better understand the biology of ATC and to test therapeutics.

**Supplementary Information:**

The online version contains supplementary material available at 10.1186/s13046-024-03009-8.

## Introduction

Anaplastic thyroid cancer (ATC) is a rare malignancy but is one of the most aggressive human cancers [1]. It remains among the most lethal diseases globally and carries a very poor prognosis [2]. The low incidence of ATC and the rapid progression of the disease have made it challenging to study its biology and to assess the effectiveness of innovative treatments through preclinical studies or clinical trials [3]. To better understand the biology of ATC, and to translate this knowledge to clinical applications, representative and robust preclinical ATC models are urgently needed.

Historically, the commonly used preclinical models were mouse models and patient-derived cell lines propagated in two-dimensional (2D) culture. However, many drawbacks hinder the applications of these models. Cancer cell lines cultured in monolayer typically fall short in reproducing the 3D organ structure and inherent tumor heterogeneity, thus having limitations in accurately representing the complexity of cancer [4]. In addition, in cancer cell extraction from tissue samples and the transition to 2D conditions, the cells undergo changes in morphology and their mechanism for cell division. The adoption of 2D culturing also contributes to the loss of a varied phenotype [5, 6]. Although patient-derived xenografts maintain the genetic and histological characteristics of the original tumors, they are often inefficient, time-consuming, and technically challenging to establish, making them impractical to use for personalized cancer therapy [7]. On the other hand, cancer cell lines do not recapitulate the solid tumor characteristics and the complex crosstalk between tumor cells and their microenvironment [4]. To overcome these limitations, researchers are currently developing novel patient-derived three-dimensional (3D) tumor culture models to reproduce the molecular complexity of a solid cancer, and to increase the suitability of testing pharmacologic agents for personalized cancer treatment.

Recently, tumor spheroid or organoid models have been widely used to model multiple human cancer types, including gastric cancer [8, 9], pancreatic cancer [10], breast cancer [11, 12], bladder cancer [13, 14], and prostate cancer [15, 16]. However, ATC spheroid models have not been systematically evaluated. Here, we describe the generation and detailed analyses of 3D models of ATC from patient-derived and immortalized ATC cell lines generated spheroid and their growth kinetics, histological architecture, gene expression, and treatment responses. These ATC spheroids maintain the key features of parental tumors and can potentially be used to select anticancer drugs for individual patients. Additionally, the successful establishment of this platform will also provide significant insights for exploring other rare cancer disease modeling potentials.

## Patient case presentation

The patient was an 80-year-old man with a history of thyroid cancer who had undergone a thyroidectomy and postoperative radioiodine ablation in the remote past. In August 2022, he was admitted to a local hospital for a rapidly expanding right neck mass and enlarged lymph nodes. A core needle biopsy of the right neck lymph node was performed and interpreted as a papillary thyroid carcinoma with features compatible with tall cell variants. A 18 F-FDG PET/CT scan showed large hypermetabolic masses in the left upper paratracheal lymph node, and multiple pulmonary nodules. In September 2022, the patient underwent a bilateral lateral and central neck node dissection and the pathology showed an anaplastic thyroid carcinoma arising out of the papillary thyroid carcinoma. *BRAF*^*V600E*^ antibody staining was positive in both the anaplastic and papillary thyroid carcinoma components of the tumor in an immunohistochemical study. Tumor samples were collected from the patient in an institutional review board-approved tissue procurement protocol.

On 11/08/2022, the patient started treatment with dabrafenib, at a dose of 150 mg twice daily, and trametinib, at 2 mg daily, and a follow-up PET/CT on 03/07/2023 showed a remarkable response to this treatment. Unfortunately, the dabrafenib and trametinib treatment was stopped due to progression of the disease shown on a PET/CT scan on 08/16/2023. Carboplatin and paclitaxel were recommended for possible disease control. However, the patient decided to not have any further treatment.

## Materials and methods

### Cell lines and culture

Human thyroid cancer cell lines 8505 C and SW1736 harboring the *BRAF*^*V600E*^ mutation were purchased from the European Collection of Cell Culture (Salisbury, United Kingdom) and Cell Lines Service GmbH (Eppelheim, Germany), respectively. *BRAF*^*Wild Type (WT)*^ cell line C643 was purchased from Cell Lines Service GmbH (Eppelheim, Germany) and THJ-16T derived from a patient with ATC was a kind gift from Dr. John A. Copland (Mayo Clinic, Jacksonville, FL). All cell lines were maintained in Dulbecco’s Eagle Medium (DMEM), supplemented with 10% fetal bovine serum (FBS), penicillin (10,000 U/mL), streptomycin (10,000 U/mL), and fungizone (250 ng/mL), in a standard humidified incubator at 37 °C in 5% CO_2_. All cell lines were authenticated by short tandem repeat profiling. Cell lines were tested for *Mycoplasma* from Idexx BioAnalytics (Columbia, MO, USA) and were negative for any contamination. S-8505 C = spheroid generated from 8505 C ATC cell line. S-C643 = spheroid generated from C643 ATC cell line.

### Human specimens

**Fig. 1:**
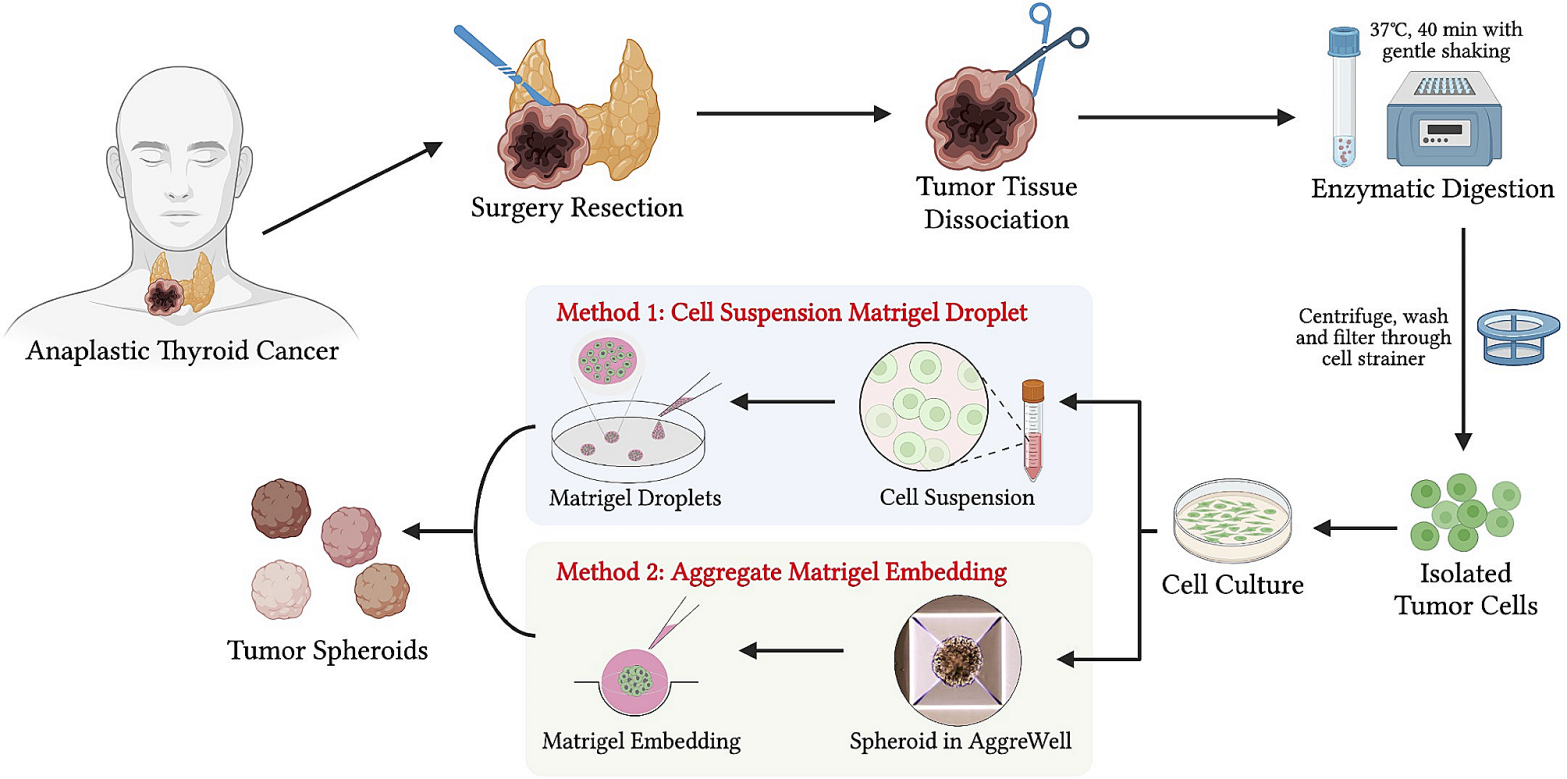
Flow diagram of establishment and characterization of ATC organoids. Generation of ATC organoid lines from patients undergoing surgery using optimized medium components, as well as characterization, RNA-sequencing, and cell proliferation assay on ATC organoids (Figure Made in BioRender.com)

Thyroid tissues were obtained from a patient who underwent surgery at Stanford Hospital. Information about the patient regarding sex, age, tumor size, and clinical stage was recorded. TNM staging was performed based on the eighth edition of the American Joint Committee on Cancer staging system. Approval for this study was obtained from the institution review board at Stanford University (approval number 50,782). The patient’s tumor tissue was procured after written informed consent had been obtained. The diagnosis was confirmed on hematoxylin-eosin (H&E)-stained slides by thyroid cancer surgical pathologists. Figure 1 summarizes the processing of the patient’s tumor samples to generate the 3D culture model.

### Tissue processing

Fresh tissue samples were immediately placed on ice, transported to the laboratory, and then washed and split into several smaller pieces. Two or three serial pieces of samples were snap-frozen and stored at -80 °C for DNA isolation, two pieces were fixed in formalin for histopathological analysis and immunohistochemistry, and the remaining tissues were dissociated and processed for spheroid derivation. Tumor tissue was finely sliced into 1–3 mm pieces with scissors. The minced tissues were digested with collagenase type II (5 mg/mL, Gibco, No. 17101-015) in the presence of the rho-associated protein kinase (ROCK) inhibitor Y-27,632 dihydrochloride (10 µM, AbMole Bioscience, No. M1817) for 20 min in a Thermo mixer at 37 °C with gentle shaking. Dissociated tissues were centrifuged at 350 g for five minutes, washed once with DMEM/F12 medium containing 15 mM HEPES and L-Glutamine (Gibco, No. 11330-032), 1% GlutaMAX (Gibco, No. 25030-081), and 1% antibiotic-Pen/strep (Gibco, No. 15140-122), and centrifuged again. The digested tissue suspension was resuspended with 5 mL of DMEM/F12 medium and filtered through a 70 μm cell strainer to remove large undigested fragments. The cell suspension was collected for primary culture. The patient sample was named ATC01. S-ATC01 = spheroid generated from ATC01 tumor sample.

### Primary cell culture and viable cell selection

Harvested cell suspensions were placed into Matrigel-precoated plates for culturing. During the first three days, cells were cultured with *Medium A* (containing DMEM/F12 medium, 15% FBS, 1% GlutaMAX, 1% antibiotic-Pen/strep, and 5 µM ROCK inhibitor Y-27,632). After three days, the culture medium was removed and a new *Medium B* was added (containing DMEM/F12 medium, 10% FBS, and 1% GlutaMAX). For the first-round passage, centrifugation was not used due to the low number of cells. Instead, the cells were split into two wells by trypsinization, and the trypsin was neutralized with 20% FBS. Regular culture *Medium B* was applied once the cells had attached to the wells and continued in culture.

### Spheroid generation using matrigel drops

The cell pellets were resuspended in ice-cold Matrigel (Corning, No. 354,230), and five to six droplets of 50 µL Matrigel-cell suspension were placed onto a preheated Petri dish, which was then placed in an incubator at 37 °C, with 5% CO_2,_ for 10 min to allow the Matrigel to solidify. The Matrigel droplets were then covered with warm complete *Medium B* and subsequently transferred into a low-attachment six-well plate (Thermo Scientific, No. 174,932) and cultured on an orbital shaker (120 rpm) at 37 ℃ incubator (5.0% CO_2_). The *Medium B* culture was changed every three days.

### Spheroid generation using AggreWell and matrigel embedding

ATC cells cultured in the flasks were dissociated into single cells and resuspended at a concentration of 2 × 10^6^ cells in 1 mL of *Medium A* per well in a 24-well AggreWell plate (STEMCELL Technologies, No. 34,415). Tumor aggregates formed at the bottom of the AggreWell after centrifugation (350 g, 5 min), and the plate was cultured in the incubator (37 °C, 5% CO_2_) overnight. On day two, an additional 500 µl of *Medium A* was added to provide a better proliferative environment. On day three, all aggregates from the AggreWell plates were collected and transferred to low-attachment six-well plates in *Medium B* (2 ml/well) and placed on an orbital shaker to provide a floating environment. On day 14 of culturing, tumor spheroids were individually embedded into the center of the Matrigel coat and transferred back to a low-attachment six-well plate for further culturing. Afterwards, Matrigel-coated ATC tumor spheroids continued to be cultured in *Medium B*. The medium was refreshed every three days.

### Tumor spheroid passage and recovery

For tumor spheroid passages, the spheroids were resuspended in 5 mL of 0.25% Trypsin-EDTA (Gibco, No. 25200-056) with incubation at 37 °C for approximately five minutes. Then they were manually shaken vigorously before the addition of DMEM/F12 (containing 10% FBS) and centrifuged at 350 g for five minutes. To maximize the development of tumor spheroids from dissociated cells, 5 µM of Y-27,632 was added during the first week of culture. Tumor spheroids were passed at a 1:2 dilution when necessary. To prepare frozen stocks, spheroids were dissociated and mixed with Recovery Cell Culture Freezing Medium (Gibco, No. 12648-010) and frozen following standard procedures [17]. When required, the tumor spheroids were thawed using standard thawing procedures and cultured as described earlier. The medium was refreshed every three days.

### Histology and immunostaining

Tumor tissues and spheroids were immersed in 10% neutral-buffered formalin for more than 24 h, embedded in paraffin, and serially sectioned at a thickness of 5 μm. The slide sections were then deparaffinized and stained with H&E and immunohistochemical markers. For immunostaining, slides were treated with a 3% hydrogen peroxide buffer for 15 min to eliminate endogenous peroxidase activity. Then, the slides were boiled for 30 min in EDTA solution (pH 8.0) for antigen retrieval and blocked in a 5% bovine serum albumin blocking buffer for 20 min at room temperature to minimize nonspecific staining. Primary antibodies against Ki-67 (1:500, Cell Signaling Technology, No. 9449), cytokeratin 19 (CK19, 1:200, Maixin Biotech, No. Kit-0030), and thyroid transcription factor 1 (TTF-1, 1:100, Cell Signaling Technology, No. 12,373) were applied to the sections and incubated at 4 °C overnight. After washing with phosphate-buffered saline (PBS), the slides were incubated with a secondary antibody at room temperature for an hour. The secondary antibodies used in the assay are anti-rabbit IgG (Invitrogen, No. 31,460) and anti-mouse IgG (Invitrogen, No. 31,430). Then, the slides were developed using 3,3-diaminobenzidine (DAB) for a duration of 30 to 60 s, and counterstained with hematoxylin, mounted, and digitally photographed using a Keyence BZ-X710 microscope (Itasca, Illinois, USA).

### RNA sequencing

RNA was isolated from the cells using a RNeasy mini kit (Qiagen, No. 74,104). A Rapid Read RNA-Seq assay was used to prepare a poly-A-enriched mRNA library from the purified RNA on the Illumina HiSeq4000 platform. *FastQC* (version 0.11.7) from the Babraham Institute was used to perform quality control checks on raw sequence data (reads in *fastq* format). The December 2013 assembly of the human reference genome (GRCh38/hg38) was obtained from the UCSC Genome Browser download site. The corresponding reference annotations (GTF) were obtained from the iGenomes site hosted by Illumina. The splice-aware aligner STAR (version 2.7.0e) was used to align reads to the human hg38 reference genome, and *SAMtools* (version 1.9) was used to index the aligned and sorted BAM files. *Cuffdiff* (Cufflinks version 2.2.1) was used for gene expression quantification (in FPKM) and for the detection of differentially expressed genes based on the BAM alignments from *STAR*. *MultiQC* (version 1.7) was run to aggregate the results from *STAR* and *FastQC* analyses across all samples into a single report. The *cummeRbund* R package (version 2.24.0 with R version 3.5.0) was used for visualization and sample clustering. *DESeq2* was used for differential expressed gene (DEG) analysis based on the negative binomial distribution [18]. The resulting *P*-values were adjusted using Benjamini and Hochberg’s approach to control the false discovery rate. Genes with an adjusted *P*-value < 0.05 as determined by *DESeq2* were assigned as differentially expressed. DEGs were visualized by *pheatmap* in R. Enrichment analyses of DEGs, using ORA (overrepresentation analysis) and gene set enrichment analysis (GSEA), was conducted using WebGestalt [19] and *clusterProfiler* [20] and visualized by *ggplot2* in R.

### Drug panel and CellTiter-Glo® 3D cell viability assay

ATC patient-derived tumor spheroids were cultured in 24-well AggreWell plates and incubated (37 °C, 5% CO_2_) for three days before treatment. The medium contained DMEM/F12 and 10% FBS. On treatment day, individual tumor spheroids were transferred into low-attachment 96-well spheroid microplates (CORNING, No. 4520) under microscope visualization, with each well containing 50 µL of medium. The following compounds were applied in triplicate per treatment at 0 ∼ 40 µM dabrafenib (Selleckchem, No. S2807), 0 ∼ 40 µM, trametinib (Selleckchem, No. S2673), and control dimethyl sulfoxide (DMSO). The drugs were diluted in DMSO according to the manufacturer’s instructions to prepare stock solutions, and then stored at -80 °C. All stock solutions were diluted to the desired concentrations with prewarmed culture medium before use.

The plates were placed on an orbital shaker to provide a floating environment and ensure adequate distribution of the drugs. Redosing was performed after three days, and the viability of the spheroids was determined after five days of treatment with the CellTiter-Glo® 3D Cell Viability Assay (Promega, No. G9682) according to the manufacturer’s instructions. Luminescence was measured using a SpectraMax® i3x plate reader (Molecular Devices).

### Statistical analyses

All data are expressed as mean ± SEM unless otherwise specified. Statistical analyses were performed using Prism GraphPad 7.0 (GraphPad Software). The significance in differences was determined by using a two-tailed Student’s *t*-test for means between two groups or by ANOVA with post hoc analysis among three more groups. Significance was set at a *P*-value of < 0.05.

## Results

### Primary culture of patient-derived ATC cells

**Fig. 2:**
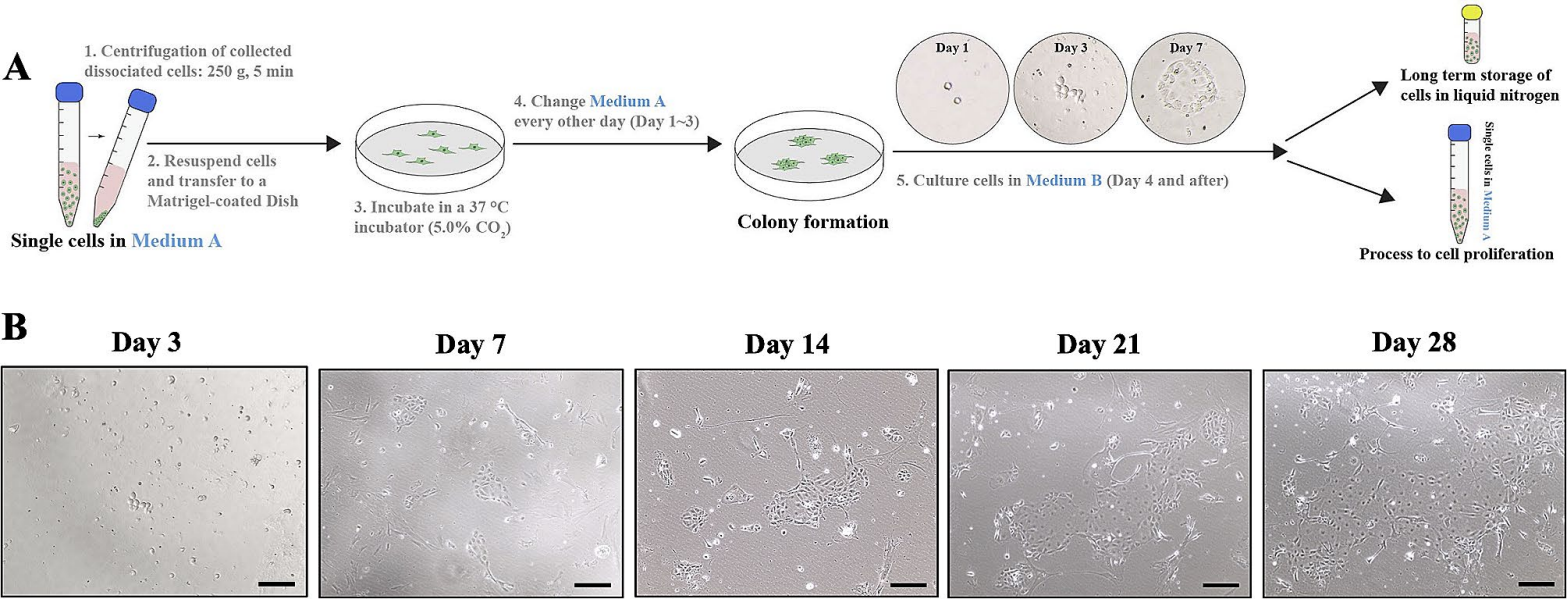
Illustration of primary ATC cell harvest and culture. (**A**) Primary ATC cells were harvested from clinical patient samples and cultured in Matrigel-coated dishes. Once there is a sufficient number of cells or colonies, cells are transferred to long-term storage in liquid nitrogen or generate tumor spheroids. (**B**) Representative checkpoint images of primary ATC cells in culture. Scale bars, 200 μm

Matrigel-coated plates were used in the study to help primary cells to attach and spread out to sufficiently absorb nutrition in the medium. Tumor tissues directly harvested from the patient were dissociated mechanically and enzymatically. After three days, small clusters of tumor cells began to attach to the Petri dish, and large colonies were observed after seven days of culture. Dissociated cells were grown in cell colonies and rapidly proliferated to reach 80% confluence after 28 days (Fig. 2).

### Establishment of ATC spheroids as matrigel droplets

**Fig. 3:**
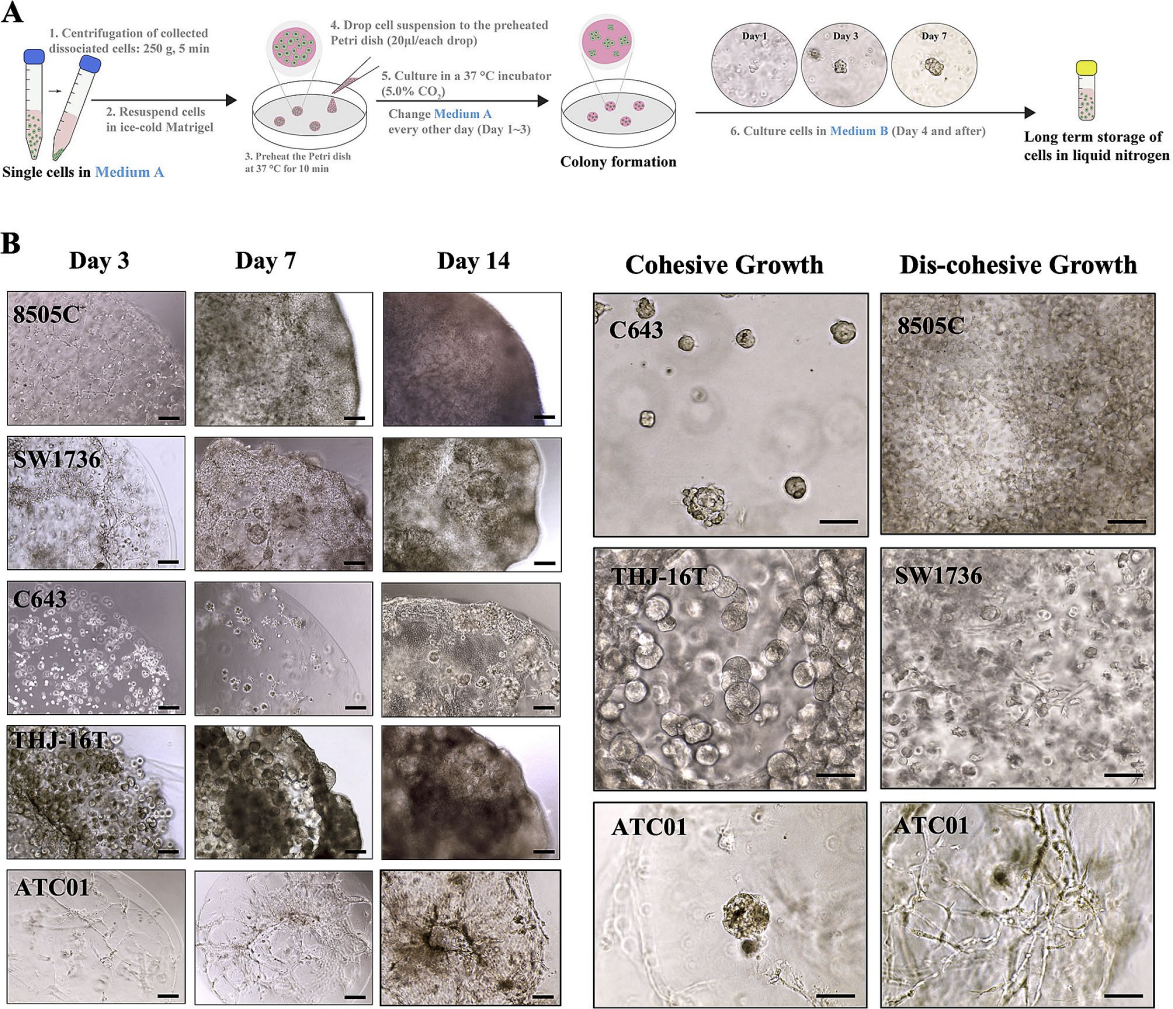
Illustration of ATC spheroid generation using Matrigel droplets and representative checkpoint images. (**A**) 3D Matrigel cell suspension droplets were used to generate ATC tumor spheroids. ATC cells were resuspended in ice-cold Matrigel liquid, then placed on a 37 ℃ preheated Petri dish as individual droplets. The tumor cell droplets were cultured in the medium and the dish was placed on an orbital shaker (120 rpm) in a 37 ℃ incubator (5.0% CO_2_); the medium was changed every one to three days. Representative images were taken at several time points. Scale bars, 200 μm. (**B**) Morphologies of developing ATC tumor spheroids generated from different human cancer cells. Left panel: representative bright-field images of ATC tumor spheroids derived from *BRAF*^*V600E*^-mutant cell lines (8505 C, SW1736), *BRAF*^*WT*^ cell lines (C6543, THJ-16T), and patient-derived cells (ATC01) throughout the time course. Right panel: high-magnification view of colony formation and cell invasion within the ATC tumor spheroid at day 14. Discohesive and cohesive growth patterns were presented in the ATC spheroids. Scale bars, 100 μm. *Medium A* = DMEM/F12 medium, 15% FBS, 1% GlutaMAX, 1% antibiotic-Pen/strep, and 5 µM ROCK inhibitor (Y-27,632); *Medium B* = DMEM/F12 medium, 10% FBS, and 1% GlutaMAX

All four ATC cell lines and the clinical sample-derived ATC cells readily formed spheroids in Matrigel droplets in culture with a unique morphology, size, and cytoskeletal organization (Fig. 3). In order to observe the formation of spheroids, a low-density single-cell suspension of ATC cells was seeded in Matrigel. Phase-contrast images taken over time allowed us to study the spheroids developmental course (Fig. 3A). We observed both cohesive (dense and solid structures) and discohesive (irregularly shaped structures) spheroids within the same culture condition across different cell lines (Fig. 3B, right panel). *BRAF*^*WT*^ ATC spheroids grew in a cohesive pattern, while *BRAF*^*V600E*^-mutant ATC spheroids had a discohesive organization. In the patient-derived spheroids, we observed both growth patterns, but mostly the discohesive type starting at day seven (Fig. 3B, left panel). Furthermore, the ATC spheroids were able to maintain the 3D structures throughout 120 days of culture (the longest time we observed).

### Establishment of ATC spheroids using AggreWell and matrigel embedding

**Fig. 4:**
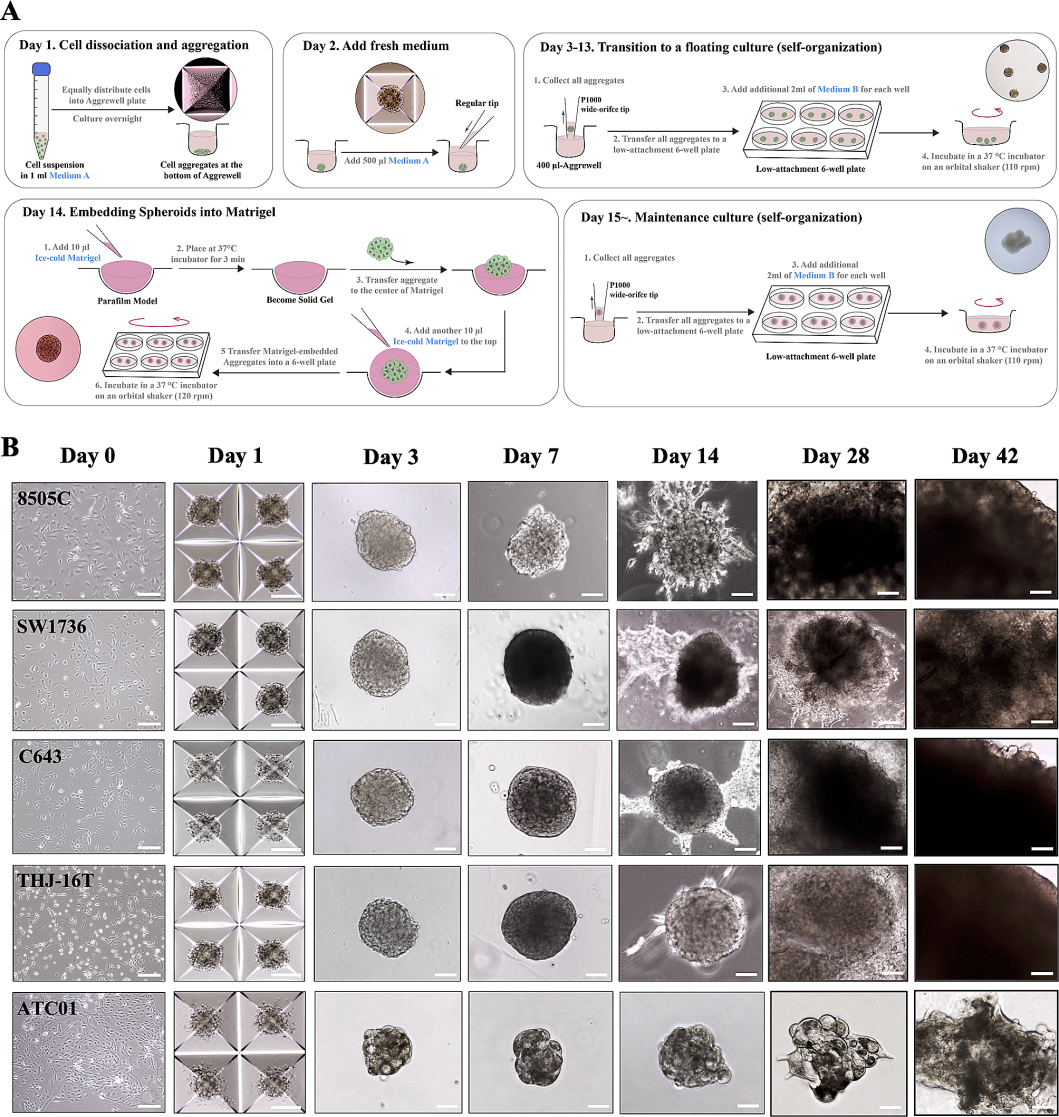
Illustration of day-by-day generation protocol and representative checkpoint images of ATC spheroids using AggreWell plate and Matrigel embedding. (**A**) Briefly, one day before the generation of tumor spheroids, the ATC cells cultured as colonies are dissociated into single cells and aggregated at a concentration of 2 × 10^6^ cells in 1 mL of *Medium A* per well in a 24-well AggreWell plate. After centrifugation, the single cells concentrate at the bottom of the AggreWell plate, forming a circular cell cluster layer. On day two, an additional 500 µl of *Medium A* is added to provide a better proliferative environment. During the process, any dead cells adhered to the aggregates are released so that the surface of the aggregates becomes clean. Starting on day three, all aggregates from the AggreWell plates are collected and transferred to low-attachment six-well plates in *Medium B* (2 ml/well) and placed on an orbital shaker to provide a floating environment. On day 14, tumor spheroids are individually embedded into the center of the Matrigel coat and transferred back to the six-well plate for further culturing. Afterwards, Matrigel-coated ATC tumor spheroids will continue to be cultured in *Medium B*. Fresh medium is changed every two to three days to provide nutrition in order to allow the continuous growth of ATC spheroids. The culture plate is placed on an orbital shaker to provide a floating environment, where the aggregates self-organize further. The medium is regularly replenished to provide sufficient nutrition during development. (**B**) Representative images are taken at 10x or 20x microscope objective magnifications as noted in each image. Scale bars, 200 μm. *Medium A* = DMEM/F12 medium, 15% FBS, 1% GlutaMAX, 1% antibiotic-Pen/strep, and 5 µM ROCK inhibitor (Y-27,632); *Medium B* = DMEM/F12 medium, 10% FBS, 1% GlutaMAX

A circular cell cluster layer was formed at the bottom of the AggreWell after centrifugation. The cells migrated and bound tightly to each other, becoming a small sphere aggregate in the center of each well by day two. Starting on day three, self-organization was initiated in response to physical and chemical cues, forming a complex structure. By day 14 of culturing, the aggregate had become rounded and condensed. Depending on the ATC cell lines, the periphery appeared wavy or linear and was visible between days 3 and 13. Thereafter, tumor spheroids were individually embedded into the center of the Matrigel coat, which provides an effective support for the attachment and differentiation of cancer cells [21–23]. ATC cells formed stable aggregates using the AggreWell plate and invaded the collagen-rich ECM after embedding (Fig. 4A). The tumor spheroids were maintained in the regular condition (Fig. 4B). However, without Matrigel embedding, the aggregates started to fall apart from day 21, most likely due to the mechanical rotational force generated by the culturing condition as plates were placed on an orbital shaker to provide a floating environment (Fig. S1).

### Recapitulation of the histopathological morphology of parental tumors in ATC spheroids

**Fig. 5:**
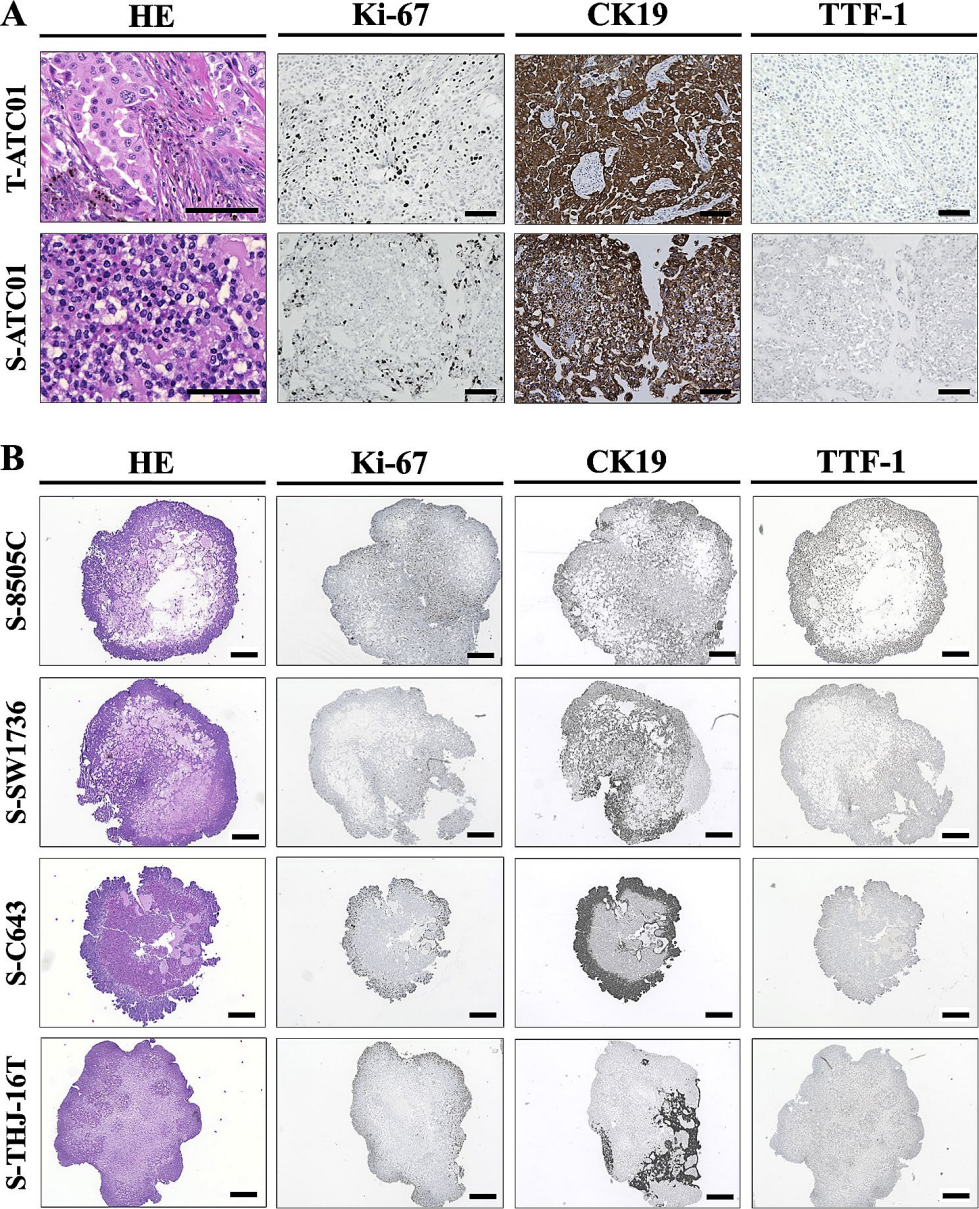
Histological characterization and marker expression analysis of anaplastic thyroid cancer (ATC) spheroids. (**A**) Hematoxylin-eosin (HE) staining of ATC01 primary tumor tissues and tumor-derived spheroids. Immunohistochemistry staining of Ki-67, cytokeratin 19 (CK19), and thyroid transcription factor 1 (TTF-1) on ATC01 primary tumor tissues and tumor-derived spheroids. T = tumor, S = spheroid. Scale bars, 100 μm. (**B**) HE staining of ATC cell line-derived tumor spheroids. Immunohistochemistry staining of Ki-67, cytokeratin 19 (CK19), and thyroid transcription factor 1 (TTF-1) on ATC cell line-derived tumor spheroids. Scale bars, 400 μm

To assess the preservation of the histological trait from the original tumors in ATC spheroids, we conducted a blinded histopathological analysis of H&E-stained sections from both tumors and spheroids. Remarkably, the histological features of tumor spheroids closely resembled those observed in the corresponding primary tumors (Fig. 5A). As expected, notable variations in the histological characteristics existed among the spheroids generated from different ATC cell lines (Fig. 5B), which is in line with other reported studies in thyroid cancer tumor spheroid models [15, 17]. The histologic findings were characterized by an admixture of anaplastic epithelial and mesenchymal components with a pleomorphic cellular population on a necrotic background. The tumor cells exhibit eccentric, oval to spindle-shaped characteristics, lack cohesive elements, and display anisocytosis features with the occurrence of multiple nuclei morphology (Fig. 5).

In addition to the preservation of the histological features, a subsequent analysis of tumor marker expression demonstrated that both ATC spheroids and their corresponding tumors displayed comparable staining patterns for Ki-67, CK19, and TTF-1 (Fig. 5A) [24, 25]. Ki-67 immunostaining revealed high cellular proliferation in spheroids and the Ki-67 positive cells were largely located in the peripheral region of the spheroids, similarly to the patient tumor sample. Cytokeratin 19 (CK19), found in normal thyroid follicular epithelium, is upregulated during neoplastic transformation and thought to be associated with thyroid cancer aggressiveness [26, 27]. In our samples, we found that CK19 was also highly expressed in both tumor tissue and derived patient spheroids (Fig. 5A). In addition, both Ki-67 and CK19 were highly expressed across four different ATC cell line-derived spheroids (Fig. 5B). Furthermore, TTF-1, a marker of thyroid differentiation, was generally negative, with only a few focally positive areas in the patient tumor sample and corresponding spheroids (Fig. 5A). These diversified expression patterns in the ATC tumor were also reported in other clinical studies, while transcription factor TTF-1 is only expressed in 5.7% of ATCs [28, 29]. Overall, these results demonstrated that tumor spheroids closely replicated the histological characteristics and marker expression patterns of their respective original tumors.

### Transcriptomic profiling of tumor spheroids

Transcriptomic analysis was performed to test whether the spheroids culture condition can more closely model in vivo tumors than monolayer cultures. We selected genes that exhibit: (1) no significant difference in DEGs between patient tumor sample and patient-derived spheroids; and (2) significant differences in DEGs when comparing patient tumor sample/spheroids with corresponding monocultures using the DESeq2 package.

**Fig. 6:**
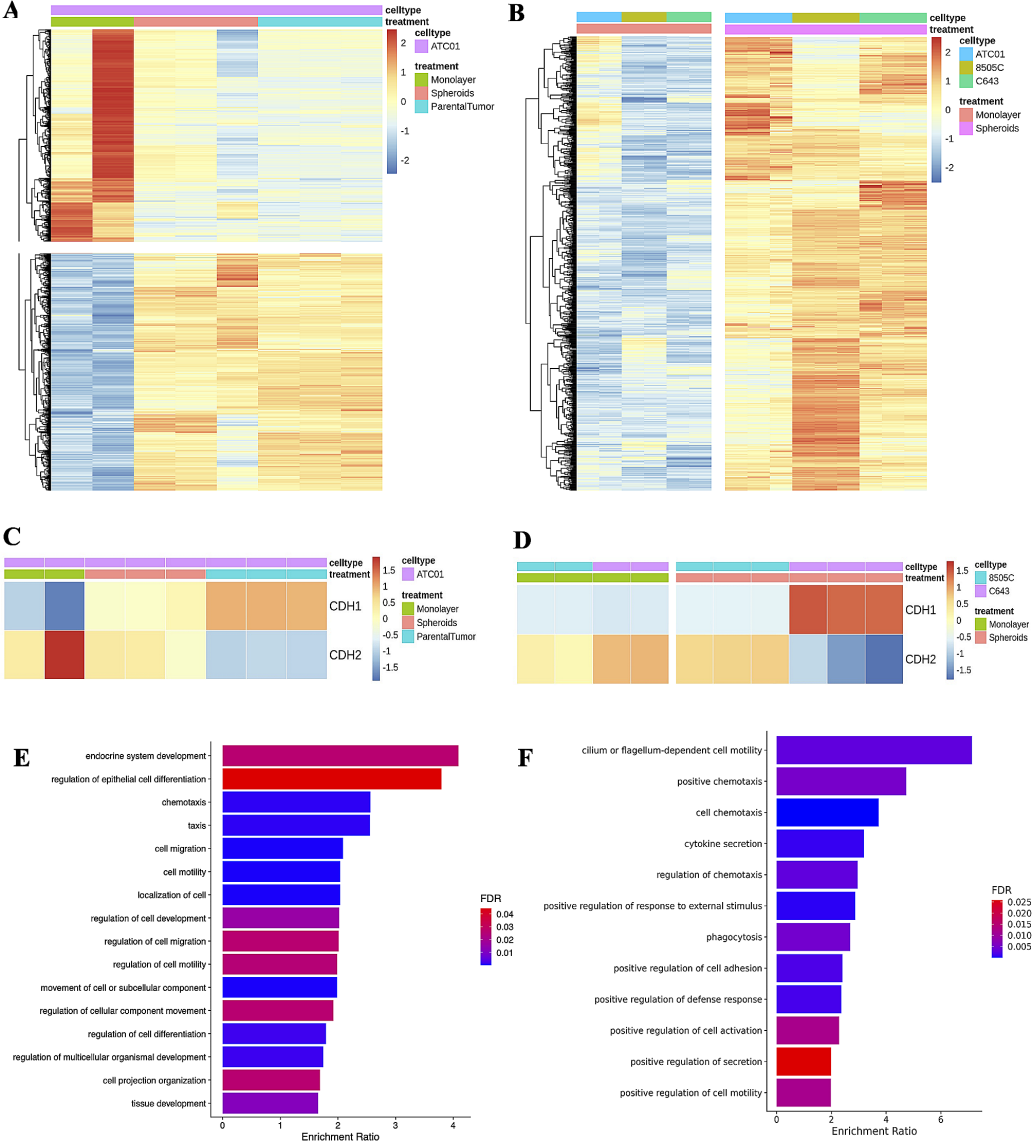
Comparison of gene expression between ATC cells from monolayer culture, spheroids culture, and parental tumor sample. (**A**) Heatmap showing gene expressions in ATC tumors and the matched monolayers and 3D spheroids. Genes with expressions that (**a**) have no significant difference between ATC tumors and matched 3D spheroids and (**b**) have a significant difference when comparing ATC tumors/matched 3D spheroids versus matched monolayers were selected. Red color represents higher expression while blue represents lower expression. (**B**) Heatmap showing upregulated gene expressions from comparison of 3D spheroids versus monolayers across 8505 C ATC cell line, C643 ATC cell line, and patient-derived ATC01 cells. (**C**) The RNA expression level of CDH1 and CDH2 in ATC01 cells. (**D**) The RNA expression level of CDH1 and CDH2 in 8505 C and C643 cells. (**E**) Pathway enrichment analysis for upregulated genes from (**A**). (**F**) Pathway enrichment analysis for upregulated genes from (**B**)

As shown in Fig. 6A, the upper panel depicts the downregulated DEGs in the ATC01 patient tumor sample and patient-derived spheroids as compared to ATC cells in monolayer culture, while the lower panel shows upregulated DEGs in the tumor sample and spheroids as compared to ATC cells in monolayer culture. The same analysis was applied to ATC cell lines 8505 C (*BRAF*^*V600E*^-mutant) and C643 (*BRAF*^*WT*^) (Fig. 6B). We found that the downregulated genes in the ATC01 monolayer cultures also had low expression in 8505 C and C643 ATC cells in monolayer culture, and upregulated genes in ATC01 spheroids demonstrated higher expression in S-8505 C and S-C643 ATC cell lines (Fig. 6B). EMT plays an important role in the invasion and metastasis of various cancers including ATC, which is associated with upregulation of CDH2/N-cadherin and downregulation of CDH1/E-cadherin [30]. We found that the mRNA expression ratio of CDH2 to CDH1 was significantly increased in monolayer cultures across all three different cell lines, including ATC01, 8505 C and C643, indicating the promotion of EMT (Fig. 6C, D left panel). However, for the ATC01 spheroids, the CDH2 to CDH1 ratio was relatively closer to the ones in the parental tumor in comparison to monolayer cultures (Fig. 6C). Interestingly, in the spheroids culturing model, cells with different genetic mutation profiles exhibited different EMT-associated gene expression profiles. As shown in Fig. 6D, the right panel exhibits the expression level of EMT-associated genes in two different ATC spheroids generated from 8505 C and C643 cell lines. Compared to *BRAF*^*WT*^ ATC spheroids (S-C643), *BRAF*^*V600E*^-mutant ATC spheroids (S-8505 C) had upregulation of CDH2/N-cadherin and downregulation of CDH1/E-cadherin, indicating the promotion of EMT in the *BRAF*^*V600E*^-mutant ATC spheroids but not in the *BRAF*^*WT*^ ones.

Pathway enrichment analysis was performed to elucidate the functional implications of the DEGs in the spheroids culture condition (Fig. 6E, F). Compared with monolayer culture, the pathways enriched in the tumor sample and patient-derived spheroids include endocrine system development, regulation of epithelial cell differentiation, chemotaxis, cell motility and projection regulations (Fig. 6E). When the same analysis was applied to all three cell lines together, very similar enriched pathways were found: cell migration, motility, adhesion, and chemotaxis. These data suggest that spheroids culture induced DEGs with common cellular functional changes that may be shared across different ATC cell lines (Fig. 6F). These findings highlight the unique capability of the spheroids model to recapitulate human-specific molecular features that could be more clinically relevant than in vitro monolayer models, especially for testing therapeutics.

### Patient-specific drug responses and correlations in respective *BRAF* mutational profiles

**Fig. 7:**
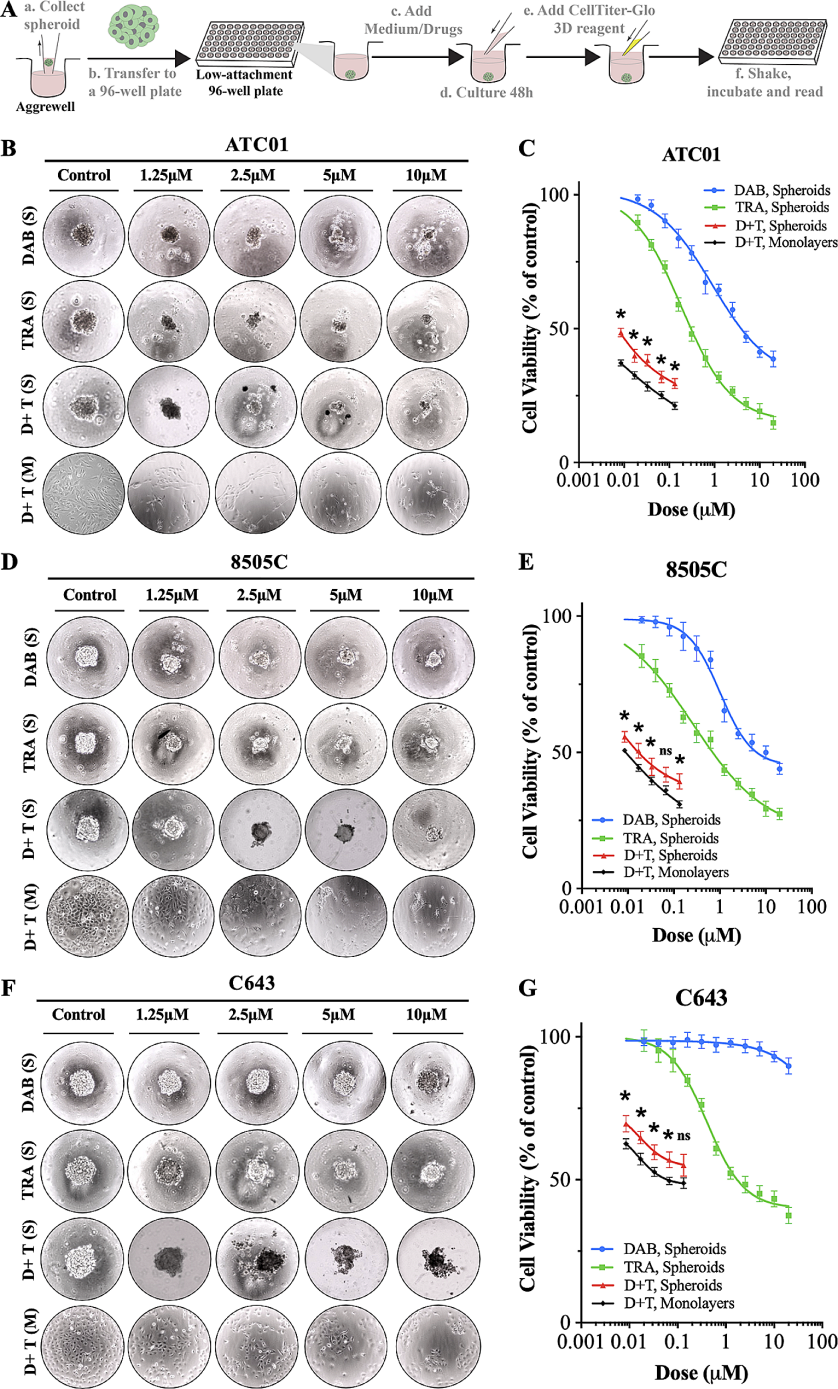
Drug responses in anaplastic thyroid cancer (ATC) spheroid lines. (**A**) Workflow for evaluating cell viability in ATC spheroids using CellTiter-Glo 3D cell viability assay. (**B**) Representative images of ATC01 spheroids and monolayers in response to different drug treatments. (**C**) Drug-response analysis with dabrafenib, trametinib, and combination of both in ATC01 spheroids and monolayer cultured cells. (**D**) Representative images of 8505 C spheroids and monolayers in response to different drug treatments. (**E**) Drug-response analysis with dabrafenib, trametinib, and combination of both in 8505 C spheroids and monolayer cultured cells. (**F**) Representative images of C643 spheroids and monolayers in response to different drug treatments. (**G**) Drug-response analysis with dabrafenib, trametinib, and combination of both in C643 spheroids and monolayer cultured cells. Cell viability was measured by CellTiter-Glo assay after five days of drug treatment, and results were normalized to dimethyl sulfoxide-treated control cells. Each data point represents the mean ± SD of three technical duplicates. *P < 0.05 is the cell viability in the spheroid models compared to the cell viability in monolayer cultured cells, ns = nonsignificant. S = spheroids; M = monolayers; DAB = dabrafenib; TRA = trametinib; D + T = dabrafenib + trametinib, the combination ratio is 150:1, the dose-response curve of the combination group was plotted based on the doses of trametinib used in the treatment. The doses for trametinib in the combination group were 0.0085 µM, 0.017 µM, 0.033 µM, 0.067 µM, 0.133 µM. Based on the combination ratio (dabrafenib:trametinib = 150:1), the doses of dabrafenib in the combination group were 1.25 µM, 2.5 µM, 5 µM, 10 µM and 20 µM, respectively

Patient tumor-derived spheroids have the potential to be used as preclinical models for evaluating the efficacy of drug compounds. In addition, the use of a combination of BRAF and MEK inhibitors has resulted in great response rates in some patients with *BRAF*^*V600E*^-mutant ATC [31]. In particular, the patient reported had an excellent initial response to combined dabrafenib and trametinib treatment. To determine whether the patient-derived tumor spheroid model would show a similar treatment response observed clinically in the patient, we performed dose-titration assays to examine the sensitivity of the tumor spheroid model to dabrafenib and trametinib treatment based on the experimental workflow (Fig. 7A). We found that S-ATC01 (S: spheroid) and S-8505 C, which are *BRAF*^*V600E*^-mutant (Fig. 7B-E), were sensitive to dabrafenib, whereas the wild type *BRAF* S-C643 was resistant (Fig. 7F, G). For selective mitogen-activated protein kinase (MEK)1/2 inhibitors, it has been shown that they can lead to growth inhibition in several cell lines containing *BRAF* mutations [32]. Similarly, we found that the sensitivity to trametinib was most pronounced in the spheroids with a *BRAF* mutant profile, S-ATC01 and S-8505 C, compared to the *BRAF* wild type S-C643 (Fig. 7B-G). In addition, the spheroids models (S-ATC01, S-8505 C, and S-C643) were significantly more resistant in response to the dabrafenib and trametinib combination therapy than the ones in monocultures consistent with the patient eventually developing resistance to the combined therapy (Fig. 7).

## Discussion

Organoid and spheroid culture models have shown enormous potential for modeling healthy and malignant tissues [33]. Tumor spheroid or organoid models are increasingly being used for various types of human cancers, including thyroid cancers [17, 34–37] However, there has been no systematic evaluation of ATC spheroid models. Our study provides a streamlined protocol for harvesting patient derived samples and the generation of spheroids. Moreover, it compared patient samples, monocultures, and spheroids, and investigated both clinical samples and ATC cell lines and their cellular, molecular, and genetic features. Additionally, the spheroid model was utilized to evaluate the efficacy of a targeted clinical treatment strategy and demonstrated that such a model better mimics response to such a clinical treatment strategy.

In this study, we described a 3D culture system for successful and efficient generation of patient-derived ATC tumor spheroid lines in vitro. 3D cultures for both patient-derived ATC and established ATC cell lines were successfully established using two methods: hanging Matrigel droplets and aggregate Matrigel embedding. We found that tumor spheroids showed closer tissue-specific architecture, cellular proliferation ability, cellular interactions and gene expression profiles than monolayer culture.

For the generation of tumor spheroids, both Matrigel droplets and aggregate Matrigel embedding techniques were able to generate stable 3D tumor derivatives. Based on our experience, the hanging Matrigel droplets method is preferred for generating tumor spheroids when the number of starting viable cells is low. This could be attributed to the function of Matrigel used in this method, as it’s enriched with extracellular matrix and growth factors, which can sustain a higher proliferation rate, similarly to the extracellular matrix presented in vivo [38]. A recent study has shown that the stable formation of uniform-sized and spherical-shaped spheroids could be generated with a starting number of as few as ten cells per spheroid when using this technique [39]. However, several disadvantages of this method have also been reported, such as the huge variations in spheroids’ diameters, limited cell viability due to direct single-cell-matrix contact and low throughput [40, 41]. Therefore, for scaling up the production of tumor spheroids, an AggreWell™ plate was used for the rapid generation of uniform cancer spheroids for ultra-high-throughput workflows [42, 43]. Thus, microwell arrays were designed to produce multiple spheroids within a single well by centrifuging single-cell suspensions to form uniform aggregates. The matrix-free method could facilitate the formation of size-controlled spheroids to improve the precision of the assay and increase the cell viability through direct cell-to-cell interaction and self-assembling [44]. In this study, we found that the ATC spheroids generated from the AggreWell™ plate started to fall apart after 21 days in culture. This could potentially be due to low levels of E-cadherin in the ATC cells, which is involved in intercellular junctions, thus limiting the ability of cells to organize themselves spontaneously into compact spheroids [45]. To overcome this limitation, these tumor cell aggregates were further encapsulated into Matrigel matrix. Similar to previous reported studies, a scaffolding extracellular environment such as Matrigel was able to facilitate the development of the microstructure architecture of tumor spheroids and closely mimic the tumor microenvironment [38, 46].

Morphologically, we observed both cohesive (dense and solid structures) and discohesive (irregularly shaped structures) spheroids within the same culture condition across different ATC cell lines. In general, *BRAF*^*WT*^ ATC spheroids tend to grow in a more cohesive pattern, while *BRAF*^*V600E*^-mutant ATC spheroids had a discohesive organization. This could be due to the decreased expression of E-cadherin in the *BRAF*^*V600E*^-mutant cells compared to *BRAF*^*WT*^ ATC cells and result in promoting cell migration and invasion [47, 48]. In our RNA sequencing data, we also found the downregulation of E-cadherin genes (CDH1) in the spheroids derived from *BRAF*^*V600E*^-mutant cells (8505 C) and not in the ones derived from *BRAF*^*WT*^ cells (C643). In the patient-derived *BRAF*^*V600E*^-mutant ATC spheroids, we observed both growth patterns but mostly the discohesive type, indicating the cellular heterogeneity in the tumor spheroids. Histologically, ATC spheroids had a similar morphology to the study patient’s tumor through H&E staining and proliferation marker staining. Characteristic features of tumor spheroids showed specific internal architecture with lumen formation, which is the result of cell apoptosis in the central part of the spheroids [49]. In line with other studies, we found that the cell proliferative markers (e.g., CK19, Ki-67) are highly expressed in the peripheral part of the 3D structures compared to the core region due to the accessibility to nutrition [50, 51]. The RNA sequencing study revealed that gene expression patterns of tumor cells derived from the spheroids closely matched parental patient tumor-derived cells in comparison to monolayer culture. Based on the DEG set, the pathways enriched in the patient-derived tumor spheroids were closely associated with the function of cancer initiation, progression, and cancer cell kinetics. Although this is the first time this has been reported in ATC tumor spheroids, better recapitulations of tumor conditions in 3D than in 2D models from the transcriptional landscape were also discovered in the modeling of other cancer diseases [52–54]. Interestingly, we also found that the genes associated with EMT were significantly upregulated in the monolayer cultured cells in comparison to the ones from parental tumor and spheroid samples. As reported, in vitro culture condition could influence the transition of epithelial cells to mesenchymal cell populations, acquiring a stem cell-like phenotype associated with malignant behavior [55–57]. Similarly, our study found that all the ATC cell lines cultured in the monolayer condition (ATC01, 8505 C, and C643) exhibited a gene expression profile that promotes EMT. This finding demonstrates that the monolayer culturing condition has the potential to obscure the in vivo tumor EMT state and may impact treatment responses when used as an in vitro model. However, for the ATC cells in spheroid culturing conditions, despite undergoing the same spheroid generation and culturing processes, cells from different cell lines exhibited a distinct EMT-associated gene expression profile. This variability can be attributed to their diverse genetic backgrounds. Furthermore, we observed comparable upregulations of CDH1 in both ATC01 spheroids and parental tumor samples, whereas no such upregulation occurred in monolayer-cultured cells, suggesting our spheroid model cultures may mimic more closely the in vivo behavior related to the EMT status. However, the limitation in our study is that we looked at one time point of culturing samples and the duration of culture may also influence EMT and the tumor microenvironment.

In addition, studies have shown that organoids and spheroids serve as more effective models for exploring drug sensitivity and resistance than cells cultured in a monolayer [58]. In our study, we found that the sensitivities to dabrafenib and trametinib were largely correlated with their *BRAF*-related mutational profiles. Remarkably, the drug kinetics in the ATC01 patient-derived spheroids closely recapitulated the drug responses seen in the patient. As reported, the tumor spheroids are typically more resistant to chemo- and radiotherapies than monocultures [59]. This was also found in our study, as ATC spheroids generated from ATC01, 8505 C, and C643 cells were more resistant to dabrafenib and trametinib combination therapy than monolayer cultured cells. Drug resistance in tumor spheroids may better predict clinical response due to several reasons, including the spheroid structure (dense or loose), cell growth state (necrotic, quiescent, proliferating cells), or the diffusion of drugs into the dense cellular/extracellular matrix structure, and enrichment of cancer stem cell population [60–62]. On the other hand, the tumor spheroid models are enriched with cancer stem cells, this may also closely associate with treatment resistance [63–65]. Ap art from drug screening, these models also allow analyses of different growth constraints such as nutrient consumption, oxygen tension, and radiation effects [66, 67].

In summary, our study is the first to report on the generation and characterization of ATC tumor spheroids. The ATC spheroids recapitulated the histologic and molecular features of the parental tumors. We believe the use of the ATC spheroid model we describe herein will pave the way for translational research and individualized therapy for patients with ATC.

### Electronic supplementary material

Below is the link to the electronic supplementary material.

**Supplementary Material 1**: **Supplementary figure S1.** Generation of ATC spheroids using AggreWell plate and cultured in the condition without Matrigel embedding. ATC cells can form stable aggregates using an AggreWell plate. They can be properly maintained in the regular condition until day 14. However, without Matrigel embedding, the aggregates start to fall apart, most likely due to the mechanical rotational force generated by the culturing condition on an orbital shaker.

**Supplementary Material 2**

## Data Availability

The datasets generated and analyzed during the current study are available on reasonable request.
