# Supplementary figures and images for: Anaplastic thyroid cancer spheroids as preclinical models to test therapeutics
Source: J Exp Clin Cancer Res. 2024 Mar 19;43:85. doi: 10.1186/s13046-024-03009-8 (PMC10949686; doi:10.1186/s13046-024-03009-8)

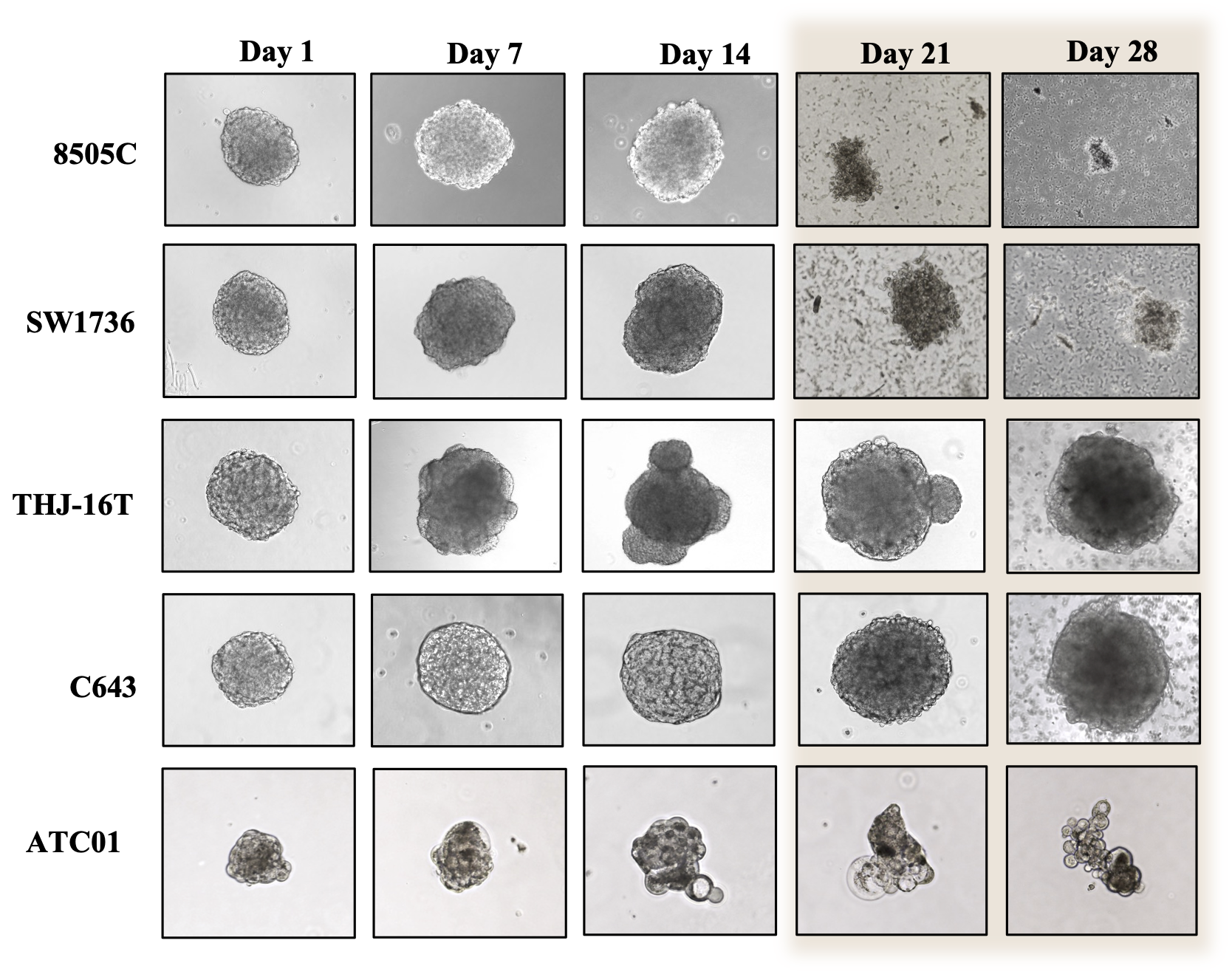

Supplement: Supplementary file 1 — Supplementary Material 1: Supplementary figure S1. Generation of ATC spheroids using AggreWell plate and cultured in the condition without Matrigel embedding. ATC cells can form stable aggregates using an AggreWell plate. They can be properly maintained in the regular condition until day 14. However, without Matrigel embedding, the aggregates start to fall apart, most likely due to the mechanical rotational force generated by the culturing condition on an orbital shaker. [file 13046_2024_3009_MOESM1_ESM.tiff]
